# Relationship of the Behavior of Older Participants with Body Composition Change: Results of the Second Wave of the Cognition of Older People, Education, Recreational Activities, Nutrition, Comorbidities, and Functional Capacity Studies (COPERNICUS)

**DOI:** 10.3390/nu15081834

**Published:** 2023-04-11

**Authors:** Agnieszka Kujawska, Guillermo F. López Sánchez, Flaka Hoti, Sławomir Kujawski, Paweł Zalewski, Kornelia Kędziora-Kornatowska

**Affiliations:** 1 Department of Exercise Physiology and Functional Anatomy, Ludwik Rydygier Collegium Medicum in Bydgoszcz, Nicolaus Copernicus University in Toruń, Świętojańska 20, 85-077 Bydgoszcz, Poland; agnieszka.kujawska@cm.umk.pl (A.K.);; 2 Division of Preventive Medicine and Public Health, Department of Public Health Sciences, School of Medicine, University of Murcia, 30120 Murcia, Spain; 3 Department of Health Management, University of Gjakova “Fehmi Agani”, 50000 Gjakova, Kosovo; 4 Laboratory of Centre for Preclinical Research, Department of Experimental and Clinical Physiology, Medical University of Warsaw, 1b Banacha Street, 02-097 Warsaw, Poland; 5 Department of Geriatrics, Collegium Medicum in Bydgoszcz, Nicolaus Copernicus University in Toruń, 85-094 Bydgoszcz, Poland

**Keywords:** diet, aging, exercise, geroprotectors, healthspan, network analysis

## Abstract

Background: To examine the relationship between the frequency of physical activities and food product consumption with body composition change after two years in a sample of older people. Methods: Body composition, mass change, frequency of physical activity, and food products consumption were measured. Depression severity, health self-assessment, cognitive function, and demographic data were included as confounders. Results: There were no significant changes in body composition except for a reduction in visceral fat level within two years (*p* < 0.05). Drinking beer and eating sweets a few times per week were associated with a significant increase in body fat percentage (*p* < 0.05). Drinking green or white tea more frequently than a few times per year was related to an increase in body fat (3.18 to 3.88%, *p* < 0.05). Contrarily, daily consumption of coffee was related to a decrease in body fat (*p* = 0.029). Subjects who ate sweets once a week or more frequently consumed coffee more often. Conclusions: More frequent drinking of beer or of green or white tea and consumption of sweets were related to an increase in body fat percentage, while daily coffee consumption was related to a decrease in body fat percentage after two years in older, healthy subjects. Noteworthily, the frequencies of food product consumption are interrelated.

## 1. Introduction

Aging is the main risk factor for most chronic disorders and conditions that decrease health span [1]. Over the past 40 years, obesity has become more common, especially among individuals 60 to 74 years old [2]. Biological mechanisms related to aging are highly complex [3]. Furthermore, it seems that aging and multiple chronic disorders share common mechanisms [1]. These might include (mal)adaptation to stress, loss of proteostasis, stem cell exhaustion, metabolism derangement, macromolecular damage, aberrant epigenetic modifications, and inflammation [4]. Obesity is linked to impairment and the aggravation of chronic conditions such as type 2 diabetes, cardiovascular disease, and osteoarthritis in older persons [5]. The inflammatory environment that is present in obesity and metabolic syndrome is present in age-related disorders including sarcopenia, frailty, and dementia as well; as a result, these two sets of pathologies feed off of one another [6,7,8,9]. 

In addition to the interrelationship of pathologies with each other, there are frequent interrelationships between two or more health behaviors of individuals that have a positive or negative effect on health depending on the type of behavior. For example, a study conducted in Japan by Yamauchi et al. (2007) [10], which included older individuals and sought to examine the relationship between physical activity and healthy dietary habits, reported a positive correlation between these two health behaviors. Thus, many studies report positive combined practices (i.e., a link between positive health behaviors), as shown in a study by Hu et al. [11], in which a high level of physical activity, adequate diet, and rest were reported in combination by a large proportion of participants. The reasons why the practice of one health behavior may influence the practice of another health behavior are not entirely clear and remain to be explored; however, self-efficacy theory is often cited as an important factor in this matter. When a person succeeds in changing one lifestyle behavior, he or she becomes more confident in practicing other healthy behaviors [12].

The network approach is an important approach to deeper identification and explanation of the various relationships. Numerous studies can be found in the literature dealing with the application of the network approach in different fields of medical sciences [13,14]. According to Havey [15], a network refers to various structures consisting of variables represented by nodes and the relationships (formally called edges) between nodes. A network refers to different structures made up of more or less interconnected variables. Therefore, networks allow us to have a deeper understanding of how interconnected variables are placed and affect an individual’s health state. Networks evolve and respond to the changes that the variables undergo, and these complex systems of connectivity between variables can only be revealed by network analysis [15].

This study aims to examine the relationship between the frequency of physical activities and food product consumption with body composition (body fat percentage, skeletal muscle, mass, and visceral fat) changes within two years in a sample of older people.

## 2. Materials and Methods

### 2.1. Study Group

A regional TV and radio marketing campaign, health-related lectures at Collegium Medicum University, several older people’s organizations in Bydgoszcz, day care facilities for the elderly, and various senior meeting groups were used to recruit participants for this study. Information on the research included a description of an opportunity to undergo free of charge physical, physiotherapeutic, nutritional, social, and cognitive examination for participants 60 years of age and older. Participants were recruited for the current study between November 2015 and February 2018.

The only exclusion criterion from the study was age under 60. The examination was conducted in the Department of Geriatrics, Collegium Medicum University Hospital in Bydgoszcz, Poland. The study was approved by the Ethics Committee, Ludwik Rydygier Memorial Collegium Medicum in Bydgoszcz, Nicolaus Copernicus University, Torun (KB 340/2015). Written, informed consent was obtained from all participants.

### 2.2. Assessment Methods

#### 2.2.1. Body Composition Analysis

Analyzing body composition and body mass was performed using the Tanita BC-545 body fat analyzer. When being weighed, participants wore light clothing. Accuracy in weighing was 0.1%. Bioelectric impedance analysis (BIA) was used to quantify the body composition indices of body fat (%), visceral fat (units), and muscle mass (kg). All parameters were measured using built-in algorithms. Whole-body fat and lean mass measurement using Tanita BC-545 have a good agreement in comparison to dual energy X-Ray absorptiometry [16]. The error of measurement using BIA might be related to nutrition status, tissue temperature, and hydration [17]. As examinations lasted for about three hours per patient, subjects were reminded to take food and drink with them and consume them if needed. In addition, an opportunity to use the toilet with a reminder was provided. Participants were instructed to stand with electrodes on their feet and their hands. Respondents provided information on their height to shorten the time spent on examination; body mass index (BMI) was calculated using WHO norms [18]. Normal body type was chosen for all participants. 

#### 2.2.2. Activity Level Questionnaire

Utilizing a previously detailed questionnaire, the frequency of current physical, mental, and social activities was evaluated [19]. Questions involved the following physical activities: short walks around the house, long walks, gymnastics, cycling, running/jogging, swimming, skiing, team games, sailing, horse riding, Nordic walking, tennis/table tennis, dancing, and work on a garden plot or mushroom collection. The following questions on the frequency of food product consumption were asked: drinking beer, green/white tea, red wine, vodka, and other 40% (80-proof) alcohols, and eating chocolate, sweets, fish, coffee, vegetables, and fruit. Frequency was coded into seven categories (“never”, “once a year”, “several times a year”, “1–2 times a month”, “once a week”, “a few times a week”, and “daily”). 

#### 2.2.3. Depression Severity and Health Assessment

The Geriatric Depression Scale (GDS), composed of 15 items, was utilized as a depression screening tool [20]. It has been demonstrated that this form can be useful in very old persons with and without cognitive impairment [21]. Questions concerned the quality of life (e.g., do you feel full of energy?), current circumstances (e.g., do you feel that your situation is hopeless?), daily activities (e.g., have you dropped many of your activities and interests?), mental health (e.g., do you feel happy most of the time?), and outlook on life (e.g., are you basically satisfied with your life?). A GDS-15 score ranges from 0 to 15 points, with a higher score reflecting greater severity of depressive symptoms 

In addition, subjects were asked to carry out a self-assessment of their current health state on a 10-point scale, with 10 meaning a perfect state of health. 

#### 2.2.4. Cognitive Function Assessment

The Montreal Cognitive Assessment (MoCA) was used to measure general cognitive function [22]. The MoCA evaluates each major cognitive function domains, including executive function, short-term memory recall, and visuospatial abilities. The former domain was examined by a mini-form of TMT B, a phonemic fluency task, and a two-item verbal abstraction task. MoCA scores range from 0 to 30 points, with a higher score denoting better global cognitive function.

#### 2.2.5. Demographic and Occupation-Related Data

A number of years spent on education was measured using a self-report questionnaire. Occupational status was at first categorized as follows: white-collar worker, a white-collar worker in a managerial position, owner of a craft/entrepreneurial enterprise, military/policeman/other uniformed service, seller/employee of trade, farmer in an individual farm, physical worker (qualified), and unskilled worker. Then, the last three categories were unified as “low occupational status” and the rest as “high occupational status”. Eventually, a dichotomous variable with the highest occupational status achieved during the course of the respondent’s career was created [19].

### 2.3. Statistical Analysis

Dependent t-tests were used to analyse whether assumptions were met; otherwise, Wilcoxon tests were used to compare results from repeated measurements (before vs. after two years). To calculate effect sizes and their confidence intervals [−95%; 95%] for dependent comparisons as well as to create violin plots, the ggstatsplot package [23] was used.

Linear regression models were performed using Jamovi [24]. Changes in the body composition indicators were treated as dependent variables. Sets of behaviours related to the frequency of physical activities or product consumption were treated as predictors. Sex (being female), presence of high occupation status, age, years of education, MoCA and GDS scores, and current health status self-assessment were used as covariates in the regression models.

Alluvial diagrams and frequency tables were created using Jamovi with the easyalluvial and ClinicoPath packages [25,26]. 

Network graphing was performed using the qgraph package [27] in R [28]. The correlation results used for creating network plot were corrected using the False Discovery Rate (FDR) with a 0.9 cut-off.

## 3. Results

Overall, 205 subjects (40 males) were examined at the baseline and re-evaluated after two years. The mean age of the participants after the two year follow-up assessment was 69.67 years (−95% CI = 68.85; 95% CI = 70.5, range 60–88). The BMI of subjects was (mean ± SD) 27.54 ± 4.44 kg/m^2^ during baseline vs. 27.45 ± 4.3 kg/m^2^ after two years. The frequencies of BMI categories are shown in Table S1. In total, 173 participants (86%) were characterized by high occupational status. Every participant had undertaken work during at least some period of their life; therefore, there were no participants in the unemployed subgroup. 

**Figure 1:**
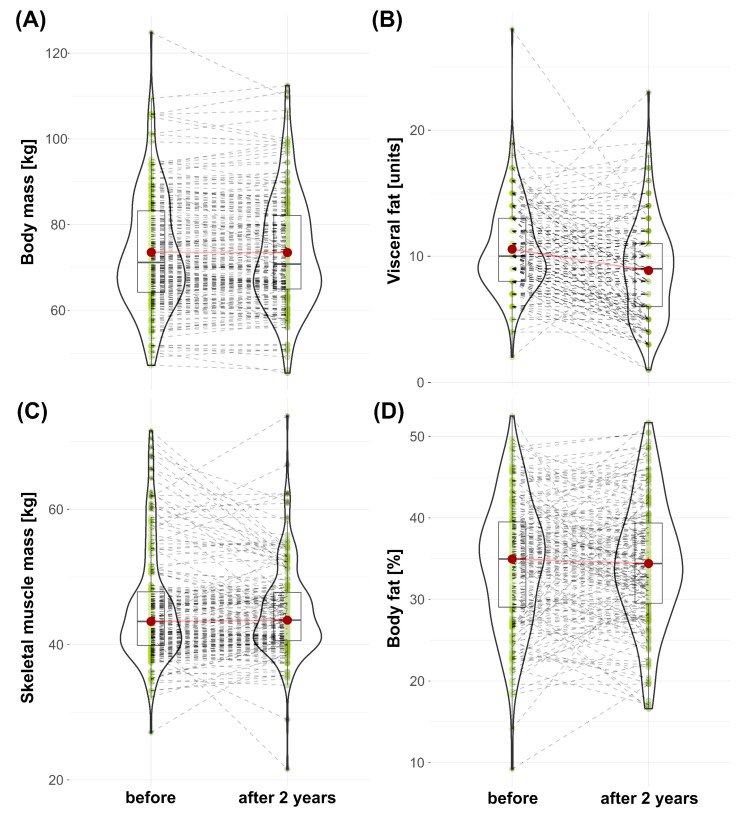
Changes in body mass and composition within two years: (**A**) change in body mass (kg) vs. after 2 years, (**B**) change in visceral fat (units) vs. after 2 years, (**C**) change in muscle mass (kg) vs. after 2 years, (**D**) change in body fat (%) vs. after 2 years. The shape of the violin graph corresponds to the distribution of values. The median value is illustrated by the horizontal black line inside the box, and the green dots connected by dashed grey lines show the results of individual participants. The arithmetical mean value is illustrated by the dark red dots connected by a light red line.

There were no significant changes in body composition within two years in the examined subjects except for the visceral fat level. No changes were observed in body weight (71.70 ± 19.03 kg during baseline vs. 70.6 ± 17 kg after 2 years, *p* = 0.758) (Figure 1A), nor were changes in muscle mass observed (43.55 ± 8.2 kg during baseline vs. 43.60 ± 7.1 kg after 2 years, *p* = 0.743) (Figure 1C). Body fat percentage did not change in a statistically significant or practically significant manner (median ± IQR (34.80 ± 10.45 % during baseline vs. 34.80 ± 9.88 % after 2 years, *p* = 0.713) (Figure 1D). Visceral fat level, on the other hand, was reduced significantly (10 ± 0.25 units during baseline vs. 9 ± 0.27 units after 2 years, *p* = 0.00000003, r = 0.55, CI 95%) (Figure 1B) [0.43, 0.66]. 

A relatively high variance in body mass changes as well as in its composition was noted; see Figure 1. Therefore, linear regression was applied to build a model to find significant predictors of body composition changes. Changes in body fat (%), skeletal muscle mass (kg), and visceral fat (units) within two years served as predicted variables. Table S2 shows the confounders included in the models. Tables S3 and S4 show a list of physical activities and diet product consumption frequency included in the regression models as dummy variables. Figures S1–S3 show the frequency and its change over a period of two years for physical activities (Figure S1), selected food products (Figure S2), and alcohol consumption (Figure S3). In the case of tennis, team games, sailing/riding on a horse, and skiing, the frequency of participants who were undertaking those activities was relatively small; therefore, these activities were omitted from further analysis. In addition, the number of categories was reduced to combine “never” with “once a year” and “several times per year” in one group. In the case of fish consumption, there were only two instances of the “daily” category; therefore, these were added to the “a few times per week” subgroup. 

Table 1 presents results from a model that explained changes in body fat percentage based on food product consumption frequency (Adjusted R^2^ = 0.17, F = 1.91, *p* = 0.003). Drinking beer a few times per week was associated with a significant increase in body fat percentage in comparison to subjects who drink beer “never” to “a few times per year” (an increase of 8.99%, *p* = 0.013). Vodka and other 40-proof alcohol and red wine consumption frequency were not significantly associated with body fat percent changes (*p* < 0.05). Subjects who consumed sweets a few times per month, once a week, a few times per week, or daily noted an increase in body fat percentage in comparison to subjects who consumed sweets ”never” to ”a few times per year” (increase in body fat of 5.41% (*p* = 0.023), 4.96% (*p* = 0.021), 4.45 % (*p* = 0.038), and 4.83% (*p* = 0.035), respectively). Participants who drank coffee a few times per week had a significantly higher increase in body fat percentage in comparison to subjects who consumed coffee daily (3.88% of body fat increase, *p* = 0.029). Subjects who consumed green or white tea ”never” to ”a few times per year” saw a decrease in body fat percentage in comparison to subjects who consumed green or white tea daily (−3.18 % of body fat, *p* = 0.028). Males gained 5.06% body fat in comparison to females (*p* < 0.001) (Table 1). 

Table S3 includes the frequency of physical activities, while Table S4 shows the frequency of food product consumption included in the regression analysis. Table S5 presents results from a model that explained changes in muscle mass in kilograms based on food product consumption frequency (Adjusted R^2^ = 0.16, F = 1.83, *p* = 0.006). Drinking beer a few times per week was related to a decrease in muscle mass in comparison to drinking beer never to a few times per year (−5.5 kg, *p* = 0.043). Sweets consumption one to two times per month was related to a decrease in muscle mass in comparison to consuming sweets never to a few times per year (−3.59 kg, *p* = 0.047). Contrarily, drinking red wine one to two times per month was related to an increase in muscle mass in comparison to drinking red wine never to a few times per year (1.88 kg, *p* = 0.049). For the rest, food product consumption frequency was not a significant predictor of changes in muscle mass within the two years (Table S2). Males lost a mean of 4.03 kg less of skeletal muscle mass in comparison to females (*p* < 0.001) (Table S5). 

Table S6 presents results from a model that explained changes in units of visceral fat based on food product consumption frequency (Adjusted R^2^ = 0.08, F = 1.37, *p* = 0.096). Food product consumption frequency was not a significant predictor of visceral fat within the two years (Table S6).

Tables S7–S9 present results from models that explained changes in body fat percentage (Table S7), muscle mass in kilograms (Table S8), and visceral fat in units (Table S9) in relation to the frequency of physical activities. The models were not statistically significant, nor were particular predictors related to physical activity frequency (*p* > 0.05). 

**Figure 2:**
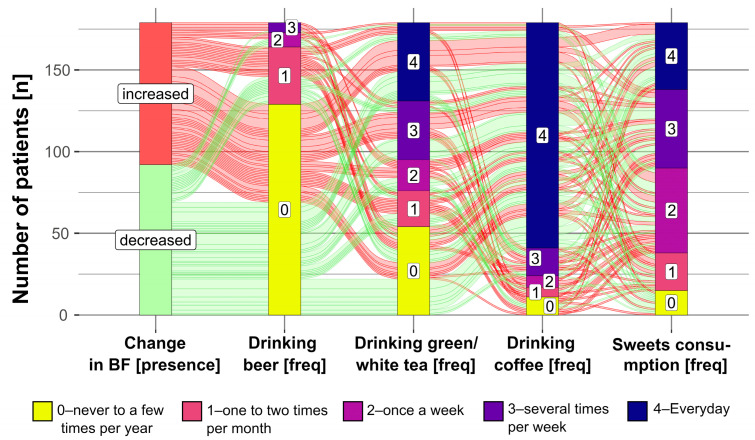
Alluvial diagram showing the relationship between the presence of body fat (BF) change with the frequency of selected food products consumption.

Figure 2 and Tables S10–S13 show the relationship between the change in body fat percentage over two years presented as a binary value (increased vs. decreased) in comparison with the frequency of food products period denoted as significant within the same in the linear regression model. Overall, older participants who noted an increase in body fat percentage within two years seemed to drink beer more frequently during this period (Table S10); 39 % of those who noted a body fat decrease consumed beer a few times per year or less frequently, compared to 33 % of participants who noted a body fact increase (Table S10). Of patients who drank green or white tea never to several times per year, n = 21 (12% of the total sample) noted an increase in body weight (Table S11). Table S12 shows that daily consumers of coffee, n = 59 (33% of the total sample) noted an increase, while n = 79 (44% of the total sample) noted a decrease in body fat percentage within two years. Table S13 shows that in participants who ate sweets never to several times per year, 6% noted a decrease in body fat percentage, while 2% noted an increase. 

In addition, as can be seen in Figure 2, the frequencies of consumption of products related to body fat percent change within two years in this study seem to be interrelated. For instance, 28 % of participants drank green or white tea a few times per year while drinking coffee daily (Table S14). Contrarily, 22 % of participants drank both beverages daily (Table S14). In addition, there seems to be a positive relationship between sweets and coffee consumption; subjects who ate sweets the most frequently (once a week or more frequently) more often consumed coffee daily (Table S15). For instance, 6 % of participants ate sweets a few times per year and drank coffee daily; however, 21%, 22%, and 21% of participants drank coffee daily and consumed sweets once a week, a few times per week, or daily, respectively (Table S15). 

**Figure 3:**
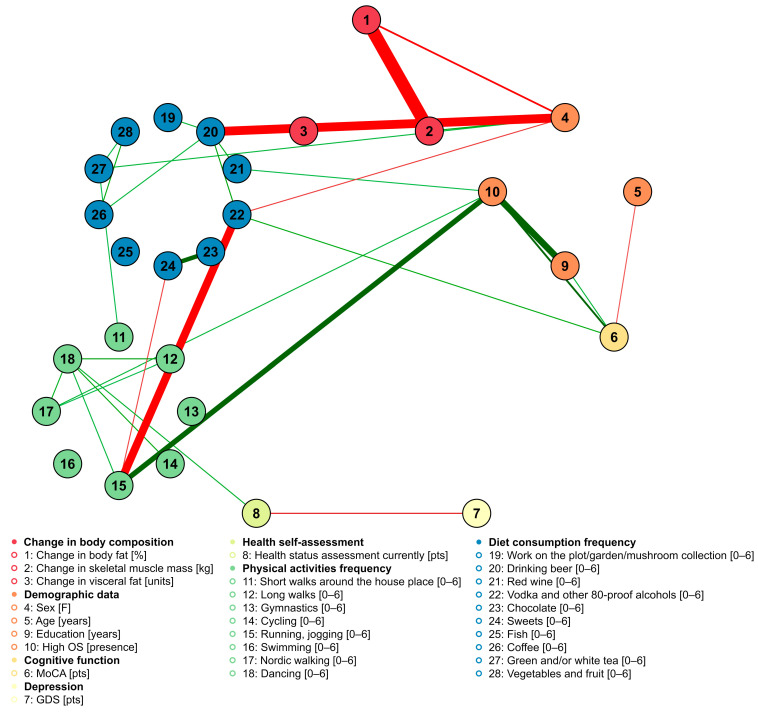
Network analysis of the relationship of body composition change with the frequency of physical activities, food products consumption, demographic data, cognitive function, depression severity, and health self-assessment. Nodes were grouped according to pre-specified groups, to which each variable belongs. Illustrated graphs are weighted, with green edges showing positive weights and red edges showing negative weights. The color saturation and the width of the edges are proportional to the absolute weight and scale relative to the strongest weight in the graph. OS—occupational status; MoCA—Montreal Cognitive Assessment; GDS—Geriatric Depression Scale.

As a part of the exploratory approach, we performed a network analysis between the examined factors (Figure 3). Interestingly, the change in the percentage of body fat was negatively related to the change in skeletal muscle mass. The created network confirmed the presence of multiple intra-group relationships meaning that there were many positive relationships between the frequency of consumption of particular food products and other food products as well as between particular physical activities and other physical activities. Interestingly, after FDR, only three edges were indicating inter-group relations between food consumption and physical activity frequency: the frequency of consumption of green tea was positively related to short walks, and the frequency of sweets consumption was negatively related to the frequency of running/jogging. A negative relationship between the frequency of drinking vodka and other 80-proof alcohols with running/jogging frequency was noted, in that a higher frequency of strong alcohol consumption was associated with a lower frequency of running (Figure 3). In addition, high occupational status was related to more frequent running/jogging, Nordic walking, and consumption of red wine (Figure 3), while being female was related to a lower frequency of beer consumption.

## 4. Discussion

In the current study, no significant changes in body weight, muscle mass, or body fat percentage were observed. However, a significant reduction in visceral fat level was noted. Changes in all body composition measurements observed within two years were characterized by relatively high variance. The frequencies of food products consumption are interrelated. For instance, subjects who ate sweets the most frequently (once a week or more frequently) consumed coffee daily more often. Such a network relationship between particular behaviours was observed in previous studies as well. For instance, Mattioli et al. noted good adherence to the Mediterranean diet and high levels of physical activity in a group of women with high coffee consumption [29]. 

### 4.1. Body Composition Trajectory in Older People

As body composition has a significant impact on disease development and physical dependence, there is great interest in understanding the progression of body composition changes to help prevent or at least mitigate them [30]. Numerous studies have evaluated or determined this progression of body composition changes, providing important results. For example, a study conducted by Visser et al. [31] aimed to assess changes in body composition in healthy older people over 60 years of age with a two-year follow-up, finding that older adults had an average change in body composition of 1–2% of total body mass (a decrease of 0.3% in men and 0.4% in women was reported), fat-free mass, appendicular lean soft tissue mass (ALST), and total fat mass after the two-year follow-up. There was a decrease in fat-free mass of 1.1% and in appendicular soft tissue mass (ASLT) of 0.8% in men, which was accompanied by an increase in total fat mass of 2.0%, while in women a decrease in body mass of 0.6% was reported after two years, with no change in ALST or body fat mass. Thus, the results of their study showed that men have a clear loss of ASLT compared to women and that men are at higher risk of loss of muscle function compared to women. Another longitudinal study conducted by Raguso et al. [30] aimed to assess body composition changes in people over 65 years of age (74 healthy men and 66 women) with a three-year follow-up period. A decrease in soft tissue lean tissue (FFST) and appendicular skeletal muscle mass (−0.3 ± 1.4 and −0.2 ± 2.2 kg, respectively) and an increase in body fat (0.6 ± 2.2 kg) were observed. 

### 4.2. Physical Activities and Product Consumption Frequency as Predictors of Skeletal Muscle Mass

In the current study, drinking beer a few times per week was related to a decrease in muscle mass in comparison to drinking beer never to a few times per year. Sweets consumption one to two times per month was related to a decrease in muscle mass in comparison to consuming sweets never to a few times per year. Contrarily, drinking red wine one to two times per month was related to an increase in muscle mass in comparison to drinking red wine never to a few times per year. The frequency of physical activities was not related to changes in body composition. Somewhat contrary to the results observed in the current study, higher muscle mass was related to a lower percentage of total and trunk fat in individuals who are [30]. Indeed, the role of physical activity and diet in maintaining lean body mass in older subjects has been underlined numerous times in the literature [32]. A recent meta-analysis showed that a resistance exercise training program was effective in inducing skeletal muscle hypertrophy in subjects 75 years of age and older [33]. 

### 4.3. Effects of Food Product Consumption Frequency on Changes in Body Composition within Two Years

In the current study, more frequent drinking of beer and of green or white tea as well as sweets consumption were related to a higher increase in body fat percentage within two years. Daily coffee consumption was related to a lower increase in body fat percentage in comparison to consumers who drank coffee a few times per week. More frequent drinking of beer and consumption of sweets was related to a decrease in skeletal muscle mass over two years. Contrarily, drinking red wine one to two times per month was related to an increase in muscle mass in comparison to drinking red wine never to a few times per year. 

In the current study, it was observed that consuming sweets a few times per month, once a week, a few times per week, or daily was related to an increase in body fat percentage in comparison to consuming sweets never up to a few times per year. In the current paradigm on weight and body composition management, it seems that the balance between caloric intake and output is the most important factor. For obese people, daily calorie restriction is a tried-and-true primary weight-loss method [34]. No significant differences have been shown in weight loss induced by a year-long low-fat diet vs. a low-carbohydrate isocaloric diet [35]. If the results of the above-described study can be replicated, then a lack of practical differences between the quality of diets with the same caloric deficit might lead to the development of a very flexible approach to obesity treatment. Presumably, if patients were allowed to choose their diet type, this could be related to specific product abundance (for instance, a relatively higher amount of carbohydrate-rich food in a low-fat diet). Such an approach might eventually lead to higher adherence to the diet, thereby increasing its efficacy.

However, it seems that the intake the certain food products and their consequences can indirectly influence the above-mentioned balance. In the current study, participants who drank coffee a few times per week had a higher increase in body fat percentage in comparison to subjects who consumed coffee daily. Subjects who consumed green or white tea never to a few times per year saw a decrease in body fat percentage in comparison to subjects who consumed green or white tea daily. Polyphenols in green tea might lead to a reduction in chronic inflammation of the liver and gastrointestinal tract and could change the gastrointestinal microbiota [36]. Modulation of the gut microbiota might in turn be related to body composition changes [37]. Contrary to our observation, tea and its components have been shown to decrease both body fat stores and body mass [36]. Presumably, there are independent mechanisms of epigallocatechin and caffeine from tea leaves that have synergistic effects on weight loss [36]. In line with our results, it has been shown that ingestion of coffee might lead to a decrease in storing of fat in the body due to inhibition of the multiplication of adipocytes, modulation of the activity of transcription factors taking part in lipid production, and the alternation of gut microbiota [36]. Nevertheless, two things should be noted about the results of the current study. First, the above-mentioned relationships between the frequency of tea and coffee consumption and body composition changes were characterized by a small effect size. Second, these relationships might in fact be spurious ones. It seems unlikely that the consumption of green or white tea or coffee has a physiologically significant effect on body composition per se. Presumably, the effects of the frequency of consumed products on body composition changes might be indirect, through modulation of gut microbiota and other mechanisms, which would eventually be related to modulation of kilocalories intake and/or expenditure, and in turn to changes in body composition. The results of previous studies have shown that decreased carbohydrate availability due to fasting or a ketogenic diet (KD), metabolic consequences of an intense physical exercise session, or impaired insulin signaling might lead to increased production of ketone bodies (KBs) [38]. KDs appear to reduce appetite, which in turn leads to a decrease in kilocalorie intake [39,40,41,42]. In addition, it has been proposed that KBs could lead to the modulation of circadian rhythms, including appetite, sleep, and hormone release [43]. Nevertheless, more studies on the potential anorexigenic effect of KD and associated mechanisms are needed [43]. Because of the potential risk of KD and limited evidence on its application in type 2 diabetes, caution in the application of KD has been advised [44]. Therefore, further studies should assess the differences between different types of diets (i.e., those low in carbohydrates vs. those relatively high in carbohydrates) with the same caloric deficit. 

### 4.4. Study Limitations

In the current study, only self-reported recall of the frequency of food product consumption and physical activity was measured, without any indication of the quantity or quality. Future longitudinal studies conducted in Poland should measure more factors related to participants’ behaviour. 

In the current study, we used a body composition measurement based on a bioelectrical impedance device. This technology has several limitations in estimating body composition with high accuracy [43]. In further studies, it would be best to accompany this method with body composition and structure measurement using dual-energy X-Ray absorptiometry or magnetic resonance imaging and waist-to-hip ratio. 

Further studies should be conducted on a larger sample size and should allow for adding more confounding factors into models, including effects of pathology, diagnosis, and treatment of chronic disorders. Characteristics of diet should be examined (plant vs. animal origin). Moreover, a larger sample might lead to higher frequencies in particular categories of qualitative variables, leading to more balanced data. For instance, it is a high need to examine the effects of unemployment and types of occupation related to no or restricted opportunities to consume regular meals during shifts, as these could be indirectly related to prolonged intermittent fasting during work time. As the R^2^ values of created models are notably small in the current study, further studies should extend the list of examined behaviors. In addition, the effect sizes of relationships between behaviors of older participants with body composition change should be provided together with a discussion on the practical and clinical significance of the results. It should be noted that behaviors denoted in the current study as statistically significant in relation to body composition change in older people may not necessarily be practically meaningful due to potential low effect sizes and/or problems with adherence of the subjects to interventions. 

As we mentioned in our previous study [19], we did not measure the method of coffee preparation in the currently analysed sample. The method used for coffee preparation might affect both the quality and quantity of substances introduced upon consumption [27,45]. Nevertheless, based on population data available in Poland, it can be predicted that the vast majority of coffee was prepared using espresso prepared under pressure in a machine or by pouring hot water over coffee (coffee in a glass or cup, without filtering, with coffee grounds) [46]. 

As highlighted in one of our previous studies, there is an urgent need for further longitudinal studies and the incorporation of a more representative sample with a higher proportion of males [19]. 

## 5. Conclusions

In the current longitudinal study, in which older participants were examined during the baseline and after two years, we have observed the following:

1. No significant changes in body weight, muscle mass, or body fat percentage were observed.
2. A significant reduction in visceral fat level was noted. Changes in all body composition measurements were characterized by relatively high variance.
3. More frequent drinking of beer, drinking of green or white tea, and consumption of sweets were related to a higher increase in body fat percentage within two years. Daily coffee consumption was related to a lower increase in body fat percentage in comparison to consumers who drank coffee a few times per week.
4. More frequent drinking of beer and consumption of sweets was related to a decrease in skeletal muscle mass within two years. Contrarily, drinking red wine one to two times per month was related to an increase in muscle mass in comparison to drinking red wine never to a few times per year.
5. Drinking beer a few times per week was related to a decrease in muscle mass in comparison to drinking beer never to a few times per year. Consuming sweets one to two times per month was related to a decrease in muscle mass in comparison to consuming sweets never to a few times per year. Contrarily, drinking red wine one to two times per month was related to an increase in muscle mass in comparison to drinking red wine never to a few times per year
6. Frequency of physical activities was not related to changes in body composition.
7. The frequencies of food product consumption are interrelated; for instance, subjects who ate sweets the most frequently (once a week or more frequently) were more often daily consumers of coffee.

## Figures and Tables

**Table 1:** Food product consumption frequencies as predictors of changes in body fat percentage within two years.

			95% Confidence Interval			
Predictor	Estimate	SE	Lower	Upper	t	*p*
Intercept ^a^	20.34	11.91	−3.22	43.89	1.71	0.090
Sex [F]:						
0–1	5.04	1.46	2.16	7.92	3.46	<0.001
High OS [presence]:						
0–1	−0.72	1.77	−4.23	2.78	−0.41	0.684
Age [years]	−0.13	0.09	−0.31	0.04	−1.50	0.136
MoCA [pts]	−0.08	0.15	−0.37	0.21	−0.53	0.596
GDS [pts]	−0.20	0.21	−0.61	0.22	−0.95	0.345
Health status assessment currently [pts]	−0.30	0.35	−0.99	0.39	−0.86	0.390
Education [years]	−0.08	0.16	−0.39	0.24	−0.47	0.638
Drinking beer [freq]:						
3–0	0.41	1.39	−2.35	3.16	0.29	0.771
4–0	0.67	2.47	−4.21	5.56	0.27	0.786
5–0	8.99	3.59	1.89	16.08	2.51	0.013
Chocolate [freq]:						
3–0	−3.01	1.77	−6.51	0.49	−1.70	0.091
4–0	−2.15	1.76	−5.62	1.32	−1.22	0.223
5–0	−3.41	1.80	−6.98	0.15	−1.89	0.060
6–0	−0.29	2.37	−4.99	4.41	−0.12	0.903
Sweets [freq]:						
3–0	5.41	2.35	0.75	10.06	2.30	0.023
4–0	4.96	2.12	0.77	9.16	2.34	0.021
5–0	4.45	2.13	0.24	8.66	2.09	0.038
6–0	4.83	2.27	0.35	9.31	2.13	0.035
Fish [freq]:						
0–4	0.80	1.64	−2.45	4.05	0.49	0.627
3–4	−0.58	1.31	−3.17	2.01	−0.44	0.658
5–4	0.27	1.54	−2.79	3.33	0.17	0.862
Coffee [freq]:						
0–6	2.85	2.08	−1.26	6.96	1.37	0.173
3–6	2.75	2.70	−2.59	8.09	1.02	0.310
4–6	−1.29	2.88	−6.99	4.41	−0.45	0.654
5–6	3.88	1.76	0.40	7.37	2.20	0.029
Green and/or white tea [freq]:						
0–6	−3.20	1.42	−6.02	−0.39	−2.25	0.026
3–6	−1.86	1.89	−5.60	1.88	−0.98	0.328
4–6	0.44	1.95	−3.42	4.30	0.23	0.821
5–6	−0.49	1.48	−3.41	2.43	−0.33	0.738
Red wine [freq]:						
3–0	−2.48	1.26	−4.97	0.01	−1.97	0.051
4–0	1.59	1.96	−2.30	5.47	0.81	0.420
5–0	−0.22	2.39	−4.95	4.51	−0.09	0.926
6–0	−0.07	5.00	−9.96	9.81	−0.01	0.988
Vegetables and fruit [freq]:						
4–3	−5.95	6.85	−19.49	7.60	−0.87	0.387
5–3	−7.49	6.57	−20.49	5.51	−1.14	0.257
6–3	−7.55	6.40	−20.21	5.11	−1.18	0.240
Vodka and other 80-proof alcohols [freq]:						
3–0	0.31	1.43	−2.52	3.13	0.21	0.831
4–0	−1.40	2.26	−5.87	3.07	−0.62	0.536
5–0	10.20	7.00	−3.64	24.05	1.46	0.147
6–0	6.58	7.07	−7.41	20.57	0.93	0.354

^a^: reference level; 0: never/once a year/several times per year; 3: 1–2 times per month; 4: once a week; 5: several times per week; 6: daily.

## Data Availability

Individual data are available from author S.K. upon request.

## Supplementary Materials

The following supporting information can be downloaded at: https://www.mdpi.com/article/10.3390/nu15081834/s1, Figure S1. Bar plots showing the relative frequency of physical activities before and after two years, Figure S2. Bar plots showing the relative frequency of selected products consumption before and after two years, Figure S3. Bar plots showing the relative frequency of alcohol consumption before and after two years, Table S1. Frequencies of BMI categories before and after two years, Table S2. List of confounding factors included in the linear regression models, Table S3. List of physical activities frequency included in the regression analysis, Table S4. List of food products consumption frequencies included in the regression analysis, Table S5. Food products consumption frequency as predictors of changes in muscle mass in kilograms within 2 years, Table S6. Food products consumption frequency as predictors of changes in visceral fat in units within 2 years, Table S7. Physical activities frequency as predictors of changes in body fat percent within 2 years, Table S8. Physical activities frequency as predictors of changes in muscle mass in kilograms within 2 years, Table S9. Physical activities frequency as predictors of changes in visceral fat in units within 2 years, Table S10. Relationship between frequency of drinking beer to the increased vs. decreased body fat, Table S11. Relationship between frequency of drinking green or white tea to the increased vs. decreased body fat, Table S12. Relationship between frequency of drinking coffee to the increased vs. decreased body fat, Table S13. Relationship between frequency of sweets consumption to the increased vs. decreased body fat, Table S14. Relationship between frequency of green/white tea and coffee consumption, Table S15. Relationship between frequency coffee and sweets consumption frequency.
