# Supplementary material for: Relationship of the Behavior of Older Participants with Body Composition Change: Results of the Second Wave of the Cognition of Older People, Education, Recreational Activities, Nutrition, Comorbidities, and Functional Capacity Studies (COPERNICUS)
Source: Nutrients. 2023 Apr 11;15(8):1834. doi: 10.3390/nu15081834 (PMC10142008; doi:10.3390/nu15081834)
Supplement: Supplementary file 1 [file nutrients-15-01834-s001.zip › nutrients-2321370-supplementary.pdf]

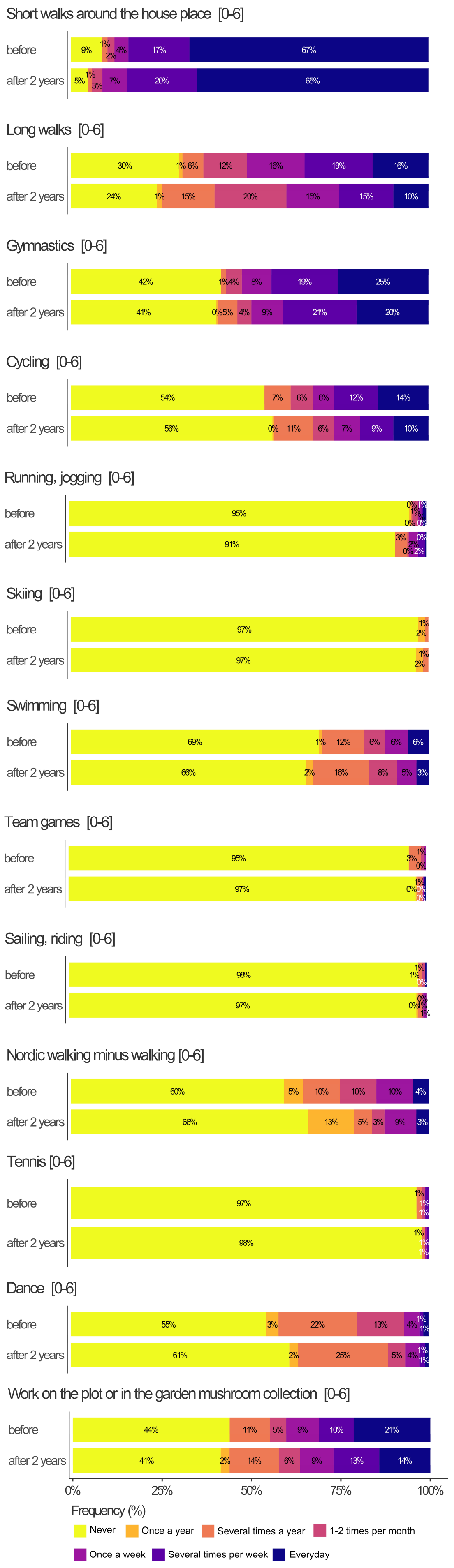

Figure S1. Bar plots showing the relative frequency of physical activities before and after two years

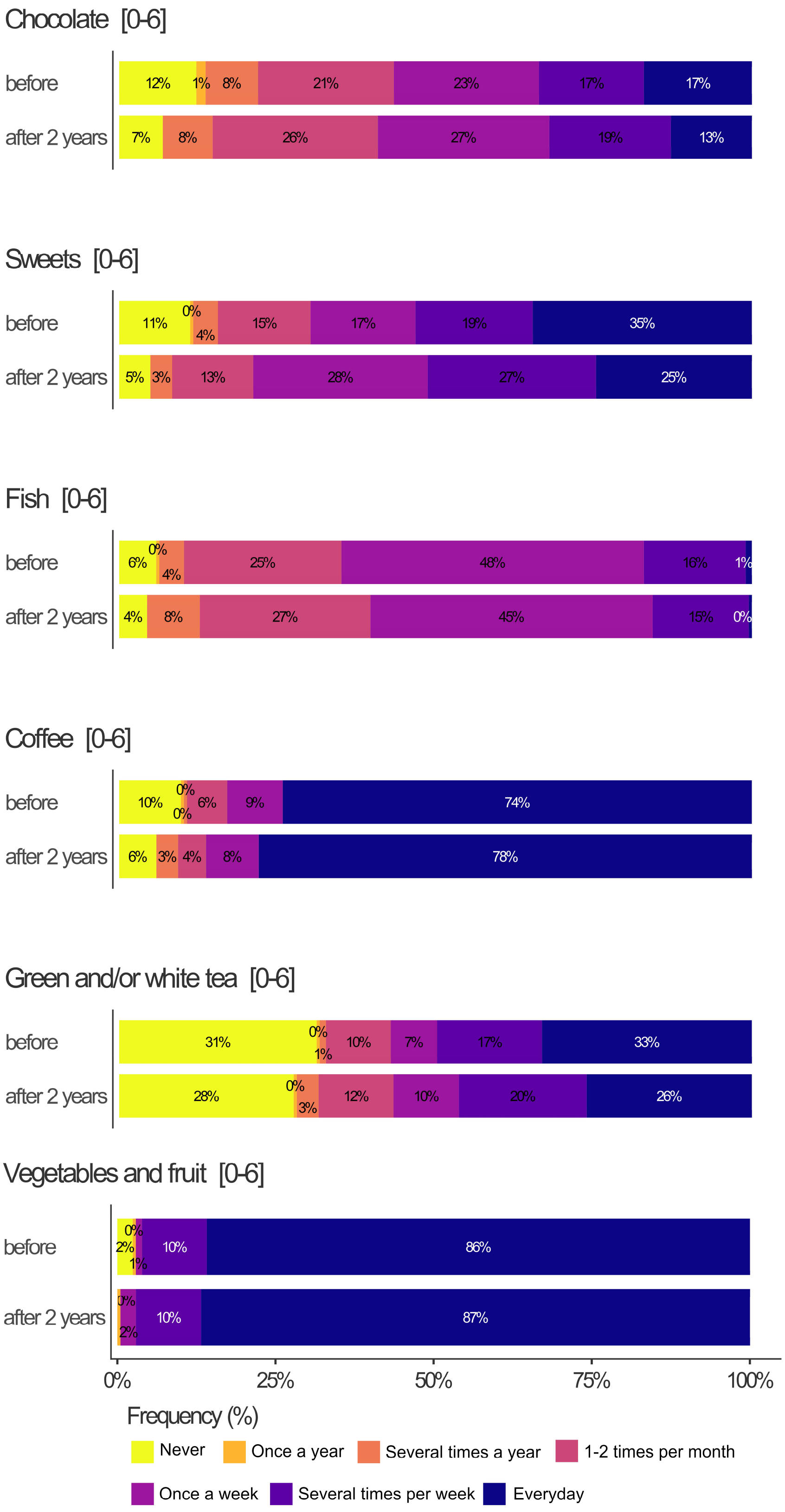

Figure S2. Bar plots showing the relative frequency of selected products consumption before and after two years

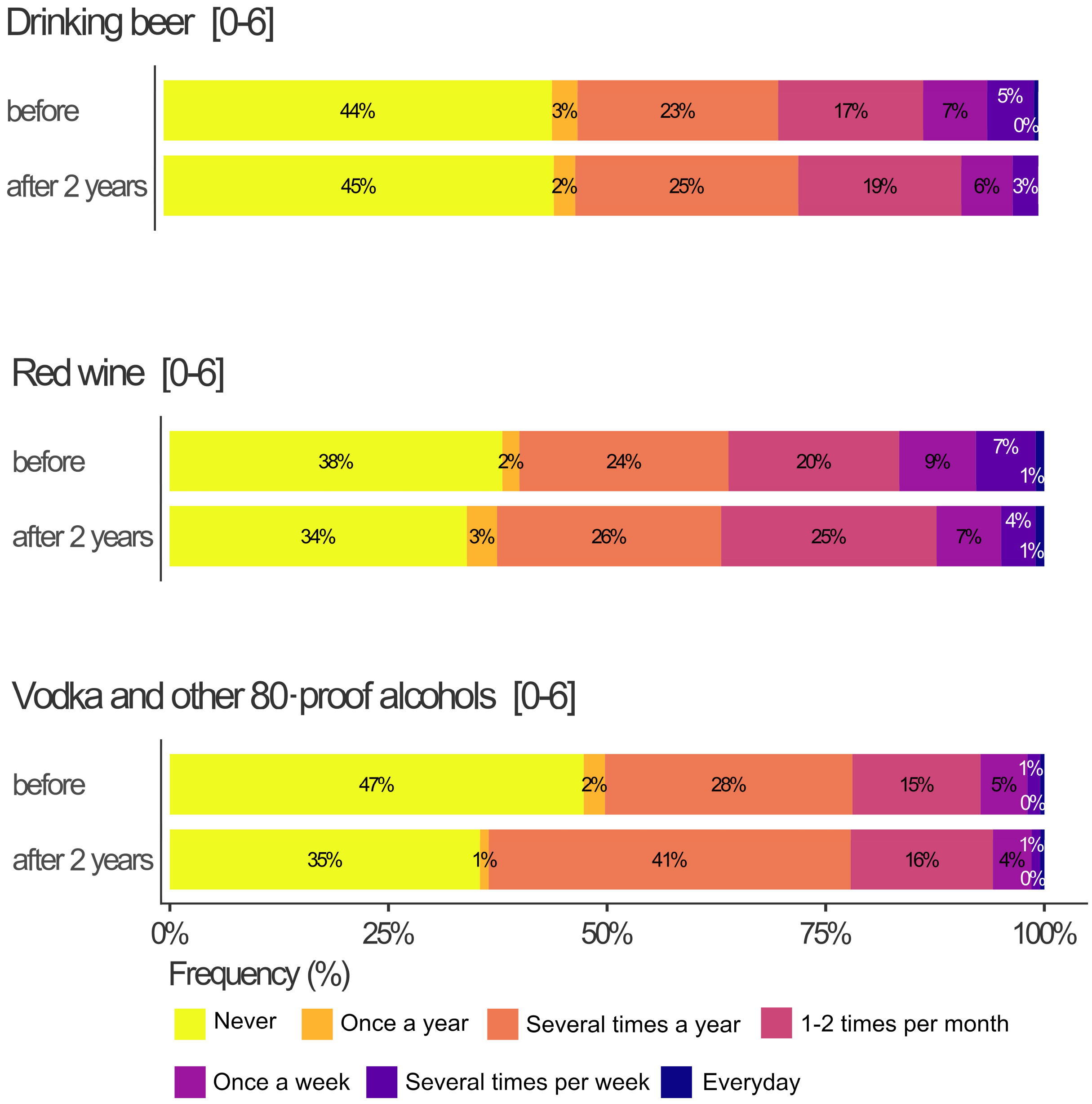

Figure S3. Bar plots showing the relative frequency of alcohol consumption before and after two years

Table S1. Frequencies of BMI categories before and after two years

BMI category	time	Counts	% of Total	Cumulative %
Underweight	before	3	1 %	1 %
	after	0	0 %	1 %
Healthy Weight	before	56	14 %	15 %
	after	64	16 %	31 %
Overweight	before	86	22 %	77 %
	after	92	23 %	100 %
Obesity	before	51	13 %	44 %
	after	47	12 %	55 %

Table S2. List of confounding factors included in the linear regression models

Predictors	Mean±SD or N (%)	The time point of measurement
Sex [female]	165 (80.5%)	baseline
Age [years]	69.67±6	after two years
GDS [pts]	3.04±2.63	after two years
Current health assessment [pts]	7.26±1.57	after two years
MoCA [pts]	23.21±4.05	after two years

Education [years]	14.25±3.5	after two years

Table S3. List of physical activities frequency included in the regression analysis

Short walks around the house place [frequency]	
Never to a few times per year	12(6%)
One to two times per month	6(3%)
Once a week	14(7%)
Several times per week	40(20%)
Everyday	132(65%)
**Long walks [frequency]**	
Never to a few times per year	82(40%)
One to two times per month	41(20%)
Once a week	30(15%)
Several times per week	31(15%)
Everyday	20(10%)
**Gymnastics [frequency]**	
Never to a few times per year	95(47%)
One to two times per month	8(4%)
Once a week	18(9%)
Several times per week	42(21%)
Everyday	41(20%)
**Cycling [frequency]**	
Never to a few times per year	138(68%)

One to two times per month	12(6%)
Once a week	15(7%)
Several times per week	19(9%)
Everyday	20(10%)
Running, jogging [frequency]	
Never to a few times per year	193(95%)
One to two times per month	1(0%)
Once a week	5(2%)
Several times per week	4(2%)
Everyday	1(0%)
Swimming [frequency]	
Never to a few times per year	170(83%)
One to two times per month	16(8%)
Once a week	11(5%)
Several times per week	7(3%)
Sailing, riding [frequency]	
Never to a few times per year	201(99%)
One to two times per month	1(0%)
Once a week	2(1%)
Nordic walking minus walking [frequency]	
Never to a few times per year	160(79%)
One to two times per month	10(5%)
Once a week	7(3%)
Several times per week	18(9%)

Everyday	7(3%)
Dancing [frequency]	
Never to a few times per year	180(89%)
One to two times per month	10(5%)
Once a week	8(4%)
Several times per week	3(1%)
Everyday	2(1%)
Work on the plot or in the garden mushroom collection [frequency]	
Never to a few times per year	117(58%)
One to two times per month	12(6%)
Once a week	19(9%)
Several times per week	26(13%)
Everyday	29(14%)

Table S4. List of food products consumption frequencies included in the regression analysis

Drinking beer [frequency]	
Never to a few times per year	148(73%)
One to two times per month	38(19%)
Once a week	12(6%)
Several times per week	6(3%)
Red wine [frequency]	
Never to a few times per year	128(63%)

One to two times per month	50(25%)
Once a week	15(7%)
Several times per week	8(4%)
Everyday	2(1%)
Vodka and other 80-proof alcohols [frequency]	
Never to a few times per year	158(78%)
One to two times per month	33(16%)
Once a week	9(4%)
Several times per week	2(1%)
Everyday	1(0%)
Chocolate [frequency]	
Never to a few times per year	30(15%)
One to two times per month	53(26%)
Once a week	55(27%)
Several times per week	39(19%)
Everyday	26(13%)
Sweets [frequency]	
Never to a few times per year	17(8%)
One to two times per month	26(13%)

Once a week	56(28%)
Several times per week	54(27%)
Everyday	50(25%)
Fish [frequency]	
Never to a few times per year	26(13%)
One to two times per month	55(27%)
Once a week	91(45%)
Several times per week	31(15%)
Everyday	1(0%)
Coffee [frequency]	
Never to a few times per year	12(6%)
One to two times per month	7(3%)
Once a week	9(4%)
Several times per week	17(8%)
Everyday	159(78%)
Green and/or white tea [frequency]	
Never to a few times per year	64(32%)
One to two times per month	24(12%)
Once a week	21(10%)
Several times per week	41(20%)
Everyday	53(26%)

	Vegetables and fruit [frequency]
One to two times per month	1(0%)
Once a week	5(2%)
Several times per week	21(10%)
Everyday	177(87%)

Table S5. Food products consumption frequency as predictors of changes in muscle mass in kilograms within 2 years

			95% Confidence Interval			
**Predictor**	**Estimate**	**SE**	**Lower**	**Upper**	**t**	***p***
Intercept [a]	-5.80	8.92	-23.45	11.86	-0.65	0.517
Sex [F]:						
0 – 1	-4.03	1.09	-6.20	-1.87	-3.69	< 0.001
High OS [presenc]e:						
0 – 1	2.22	1.37	-0.49	4.94	1.62	0.108
Age [years]	-0.00	0.07	-0.14	0.13	-0.06	0.950
MoCA [pts]	0.14	0.11	-0.08	0.36	1.29	0.199
GDS [pts]	-0.14	0.16	-0.45	0.17	-0.91	0.366
Health status assessment currently [pts]	-0.14	0.27	-0.66	0.39	-0.51	0.608
Education [years]	0.05	0.12	-0.19	0.29	0.41	0.685
Drinking beer [freq]:						
3 – 0	0.22	1.04	-1.84	2.29	0.21	0.830
4 – 0	0.27	1.85	-3.39	3.94	0.15	0.883
5 – 0	-5.50	2.69	-10.81	-0.18	-2.05	0.043
Chocolate [freq]:						
3 – 0	1.52	1.33	-1.12	4.15	1.14	0.257
4 – 0	0.98	1.33	-1.65	3.60	0.74	0.463
5 – 0	0.82	1.35	-1.85	3.49	0.61	0.545
6 – 0	0.72	1.78	-2.80	4.24	0.41	0.685
Sweets [freq]:						
3 – 0	-3.59	1.79	-7.12	-0.05	-2.01	0.047
4 – 0	-2.37	1.59	-5.51	0.78	-1.49	0.139
5 – 0	-0.96	1.60	-4.11	2.20	-0.60	0.550
6 – 0	-1.82	1.70	-5.18	1.54	-1.07	0.285

Fish [freq]:						
0 – 4	-1.12	1.23	-3.56	1.32	-0.91	0.366
3 – 4	-0.73	0.98	-2.67	1.21	-0.75	0.456
5 – 4	2.01	1.16	-0.28	4.30	1.74	0.085
Coffee [freq]:						
0 – 6	-0.95	1.56	-4.03	2.13	-0.61	0.544
3 – 6	-1.60	2.03	-5.61	2.41	-0.79	0.433
4 – 6	1.83	2.22	-2.55	6.21	0.83	0.410
5 – 6	-2.53	1.32	-5.15	0.08	-1.91	0.058
Green and/or white tea [freq]:						
0 – 6	1.29	1.07	-0.82	3.40	1.21	0.230
3 – 6	0.72	1.42	-2.08	3.53	0.51	0.612
4 – 6	-1.82	1.46	-4.71	1.08	-1.24	0.216
5 – 6	-0.30	1.11	-2.50	1.89	-0.27	0.785
Red wine [freq]:						
3 – 0	1.88	0.95	0.00	3.76	1.98	0.049
4 – 0	-1.18	1.47	-4.09	1.73	-0.80	0.423
5 – 0	-1.77	1.79	-5.31	1.78	-0.99	0.326
6 – 0	-2.50	3.75	-9.91	4.91	-0.67	0.506
Vegetables and fruit [freq]:						
4 – 3	2.86	5.24	-7.51	13.23	0.55	0.587
5 – 3	3.33	4.93	-6.41	13.08	0.68	0.500
6 – 3	4.63	4.80	-4.86	14.11	0.96	0.337
Vodka and other 80-proof alcohols [freq]:						
3 – 0	-0.20	1.08	-2.34	1.94	-0.18	0.854
4 – 0	1.27	1.69	-2.08	4.62	0.75	0.454

5 – 0	-8.12	5.25	-18.50	2.26	-1.55	0.124
6 – 0	-2.56	5.32	-13.07	7.96	-0.48	0.631

[a]: reference level, 0: never-once a year-several times per year, 3: 1-2 times per month, 4: once a week, 5: several times per week, 6: daily

Table S6. Food products consumption frequency as predictors of changes in visceral fat in units within 2 years

			95% Confidence Interval			
**Predictor**	**Estimate**	**SE**	**Lower**	**Upper**	**t**	***p***
Intercept [a]	-3.18	7.51	-18.04	11.67	-0.42	0.672
Sex [F]:						
0 – 1	-1.82	0.92	-3.64	-0.00	-1.98	0.050
High OS [presence]:						
0 – 1	0.74	1.15	-1.55	3.02	0.64	0.525
Age [years]	-0.04	0.06	-0.15	0.08	-0.64	0.523
MoCA [pts]	0.16	0.09	-0.03	0.34	1.67	0.097
GDS [pts]	-0.03	0.13	-0.29	0.23	-0.21	0.834
Health status assessment currently [pts]	0.15	0.22	-0.30	0.59	0.66	0.511
Education [years]	0.11	0.10	-0.09	0.31	1.08	0.281
Drinking beer [freq]:						
3 – 0	1.24	0.88	-0.49	2.97	1.42	0.159
4 – 0	1.05	1.56	-2.03	4.13	0.67	0.501
5 – 0	1.36	2.26	-3.11	5.82	0.60	0.548
Chocolate [freq]:						
3 – 0	-0.61	1.14	-2.85	1.64	-0.53	0.594
4 – 0	-0.08	1.13	-2.31	2.16	-0.07	0.947
5 – 0	-0.06	1.14	-2.31	2.20	-0.05	0.960
6 – 0	1.70	1.51	-1.28	4.68	1.13	0.262
Sweets [freq]:						
3 – 0	-0.21	1.51	-3.19	2.77	-0.14	0.889
4 – 0	0.61	1.34	-2.05	3.26	0.45	0.652

5 – 0	0.27	1.34	-2.39	2.93	0.20	0.842
6 – 0	-0.52	1.43	-3.35	2.31	-0.37	0.715
Fish [freq]:						
0 – 4	1.85	1.07	-0.26	3.96	1.73	0.085
3 – 4	0.42	0.82	-1.21	2.05	0.51	0.609
5 – 4	0.73	0.97	-1.20	2.65	0.75	0.456
Coffee [freq]:						
0 – 6	1.29	1.31	-1.29	3.88	0.99	0.325
3 – 6	0.93	1.70	-2.44	4.29	0.55	0.586
4 – 6	-0.31	1.86	-3.99	3.37	-0.17	0.868
5 – 6	2.57	1.11	0.38	4.77	2.32	0.022
Green and/or white tea [freq]:						
0 – 6	0.57	0.90	-1.21	2.36	0.63	0.527
3 – 6	-0.58	1.20	-2.96	1.79	-0.48	0.630
4 – 6	1.63	1.24	-0.81	4.08	1.32	0.189
5 – 6	1.66	0.94	-0.19	3.51	1.77	0.079
Red wine [freq]:						
3 – 0	-0.61	0.81	-2.22	1.00	-0.75	0.457
4 – 0	-1.45	1.23	-3.89	0.99	-1.18	0.241
5 – 0	-0.16	1.50	-3.14	2.81	-0.11	0.915
6 – 0	3.32	3.15	-2.91	9.55	1.05	0.294
Vegetables and fruit [freq]:						
4 – 3	-0.59	4.40	-9.29	8.11	-0.13	0.893
5 – 3	-4.01	4.13	-12.18	4.17	-0.97	0.335
6 – 3	-3.45	4.02	-11.41	4.51	-0.86	0.393
Vodka and other 80-proof alcohols [freq]:						

3 – 0	-1.01	0.91	-2.82	0.79	-1.11	0.269
4 – 0	3.95	1.50	0.97	6.92	2.63	0.010
5 – 0	-4.19	4.40	-12.90	4.52	-0.95	0.343
6 – 0	4.24	4.46	-4.59	13.07	0.95	0.344

[a]*: reference level, 0: never-once a year-several times per year, 3: 1-2 times per month, 4: once a week, 5: several times per week, 6: daily*

Table S7. Physical activities frequency as predictors of changes in body fat percent within 2 years

			95% Confidence Interval			
**Predictor**	**Estimate**	**SE**	**Lower**	**Upper**	**t**	***p***
Intercept [a]	7.74	10.35	-12.73	28.21	0.75	0.456
Sex [Female]:						
0 – 1	5.43	1.47	2.51	8.35	3.68	< 0.001
Age [years]	-0.08	0.10	-0.27	0.12	-0.76	0.447
MoCA [pts]	-0.05	0.15	-0.34	0.24	-0.33	0.739
GDS [pts]	-0.23	0.24	-0.71	0.24	-0.98	0.327
Health status assessment currently [pts]	-0.30	0.40	-1.10	0.50	-0.75	0.455
Education [years]	-0.15	0.19	-0.53	0.24	-0.76	0.447
High OS [presence]:						
0 – 1	-1.64	1.86	-5.32	2.04	-0.88	0.379
Short walks around the house place [freq]:						
3 – 0	3.20	4.15	-5.01	11.40	0.77	0.442
4 – 0	4.98	3.25	-1.44	11.41	1.53	0.127
5 – 0	0.93	2.78	-4.56	6.42	0.33	0.739
6 – 0	2.10	2.48	-2.81	7.01	0.85	0.399
Long walks [freq]:						
3 – 0	0.76	1.54	-2.28	3.80	0.50	0.621
4 – 0	0.52	1.92	-3.28	4.31	0.27	0.788
5 – 0	1.00	1.80	-2.57	4.56	0.55	0.580
6 – 0	2.04	2.01	-1.94	6.02	1.02	0.312
Gymnastics [freq]:						
3 – 0	2.89	2.95	-2.94	8.72	0.98	0.328

4 – 0	-1.69	2.31	-6.25	2.88	-0.73	0.466
5 – 0	-0.75	1.60	-3.92	2.43	-0.47	0.643
6 – 0	0.44	1.48	-2.50	3.37	0.29	0.770
Cycling [freq]:						
3 – 0	2.73	2.69	-2.60	8.07	1.01	0.312
4 – 0	2.35	2.57	-2.74	7.44	0.91	0.363
5 – 0	-1.66	2.01	-5.64	2.31	-0.83	0.409
6 – 0	-2.07	2.02	-6.06	1.92	-1.03	0.306
Running, jogging [freq]:						
3 – 0	-7.47	8.12	-23.53	8.59	-0.92	0.359
4 – 0	0.47	3.45	-6.35	7.29	0.14	0.892
5 – 0	-4.43	4.95	-14.23	5.38	-0.89	0.373
6 – 0	7.39	6.82	-6.11	20.88	1.08	0.281
Swimming [freq]:						
3 – 0	4.30	2.32	-0.29	8.90	1.85	0.066
4 – 0	0.59	2.30	-3.97	5.15	0.25	0.799
5 – 0	1.13	3.30	-5.40	7.66	0.34	0.733
Nordic walking minus walking [freq]:						
3 – 0	-0.16	2.88	-5.85	5.54	-0.05	0.957
4 – 0	3.22	2.97	-2.66	9.10	1.08	0.280
5 – 0	0.89	2.22	-3.51	5.29	0.40	0.689
6 – 0	-1.13	2.91	-6.89	4.62	-0.39	0.698
Dancing [freq]:						
3 – 0	-0.12	2.46	-4.98	4.74	-0.05	0.961
4 – 0	1.43	3.10	-4.71	7.57	0.46	0.645
5 – 0	-2.47	4.72	-11.81	6.87	-0.52	0.602
6 – 0	8.70	5.71	-2.60	20.00	1.52	0.130

Work on the plot or in the garden mushroom collection [freq]:						
3 – 0	-1.25	2.44	-6.09	3.58	-0.51	0.608
4 – 0	0.10	1.90	-3.66	3.86	0.05	0.956
5 – 0	2.02	1.83	-1.60	5.64	1.10	0.272
6 – 0	-1.38	1.96	-5.25	2.49	-0.70	0.482

[a]: reference level, 0: never-once a year-several times per year, 3: 1-2 times per month, 4: once a week, 5: several times per week, 6: daily

Table S8. Physical activities frequency as predictors of changes in muscle mass in kilograms within 2 years

			95% Confidence Interval			
**Predictor**	**Estimate**	**SE**	**Lower**	**Upper**	**t**	***p***
Intercept [a]	-2.94	7.08	-16.94	11.07	-0.42	0.679
Sex [Female]:						
0 – 1	-4.55	1.01	-6.55	-2.56	-4.51	< 0.001
Age [years]	-0.03	0.07	-0.16	0.11	-0.38	0.705
MoCA [pts]	0.11	0.10	-0.09	0.31	1.09	0.280
GDS [pts]	0.07	0.16	-0.25	0.40	0.44	0.659
Health status assessment currently [pts]	0.13	0.28	-0.43	0.68	0.45	0.652
Education [years]	0.09	0.13	-0.17	0.35	0.67	0.503
High OS [presence]:						
0 – 1	3.25	1.30	0.68	5.81	2.50	0.014
Short walks around the house place [freq]:						
3 – 0	-1.48	2.84	-7.10	4.14	-0.52	0.604
4 – 0	0.02	2.22	-4.37	4.41	0.01	0.993
5 – 0	0.27	1.90	-3.48	4.03	0.14	0.885
6 – 0	-0.38	1.70	-3.74	2.98	-0.22	0.824
Long walks [freq]:						
3 – 0	0.55	1.05	-1.53	2.63	0.52	0.604
4 – 0	1.47	1.33	-1.17	4.11	1.10	0.273
5 – 0	1.53	1.23	-0.91	3.96	1.24	0.218
6 – 0	0.94	1.37	-1.78	3.66	0.69	0.494
Gymnastics [freq]:						
3 – 0	-1.54	2.02	-5.53	2.45	-0.76	0.447

4 – 0	-0.18	1.58	-3.30	2.94	-0.11	0.911
5 – 0	0.18	1.10	-1.99	2.35	0.17	0.869
6 – 0	0.06	1.02	-1.96	2.07	0.06	0.955
Cycling [freq]:						
3 – 0	-2.06	1.84	-5.71	1.59	-1.12	0.266
4 – 0	-2.29	1.76	-5.77	1.19	-1.30	0.195
5 – 0	1.07	1.37	-1.65	3.79	0.78	0.436
6 – 0	0.26	1.43	-2.57	3.10	0.18	0.854
Running, jogging [freq]:						
3 – 0	10.26	5.56	-0.75	21.26	1.84	0.068
4 – 0	4.33	2.36	-0.33	9.00	1.84	0.068
5 – 0	1.61	3.39	-5.09	8.31	0.47	0.636
6 – 0	-2.73	4.66	-11.96	6.50	-0.59	0.559
Swimming [freq]:						
3 – 0	-3.08	1.59	-6.23	0.07	-1.93	0.055
4 – 0	0.25	1.58	-2.87	3.36	0.16	0.876
5 – 0	-0.83	2.27	-5.31	3.66	-0.36	0.716
Nordic walking minus walking [freq]:						
3 – 0	-1.53	1.97	-5.43	2.37	-0.78	0.438
4 – 0	0.78	2.03	-3.24	4.79	0.38	0.703
5 – 0	-1.16	1.53	-4.18	1.86	-0.76	0.448
6 – 0	-1.11	1.99	-5.05	2.82	-0.56	0.576
Dancing [freq]:						
3 – 0	-1.19	1.68	-4.52	2.14	-0.71	0.480
4 – 0	-2.44	2.12	-6.63	1.76	-1.15	0.253
5 – 0	1.27	3.23	-5.12	7.65	0.39	0.695
6 – 0	-3.45	3.92	-11.21	4.31	-0.88	0.381

Work on the plot or in the garden mushroom collection [freq]:						
3 – 0	-0.35	1.67	-3.65	2.96	-0.21	0.836
4 – 0	-0.26	1.30	-2.83	2.32	-0.20	0.845
5 – 0	-0.27	1.25	-2.75	2.20	-0.22	0.828
6 – 0	1.83	1.37	-0.87	4.54	1.34	0.183

[a]: reference level, 0: never-once a year-several times per year, 3: 1-2 times per month, 4: once a week, 5: several times per week, 6: daily

Table S9. Physical activities frequency as predictors of changes in visceral fat in units within 2 years

			95% Confidence Interval			
**Predictor**	**Estimate**	**SE**	**Lower**	**Upper**	**t**	**p**
Intercept [a]	-2.43	6.26	-14.83	9.96	-0.39	0.698
Sex [Female]:						
0 – 1	-1.66	0.89	-3.42	0.10	-1.86	0.065
Age [years]	-0.05	0.06	-0.17	0.07	-0.90	0.368
MoCA [pts]	0.12	0.09	-0.06	0.30	1.37	0.174
GDS [pts]	0.01	0.14	-0.28	0.29	0.05	0.960
Health status assessment currently [pts]	-0.02	0.25	-0.51	0.47	-0.09	0.932
Education [years]	0.23	0.12	-0.01	0.46	1.92	0.057
High OS [presence]:						
0 – 1	1.12	1.15	-1.15	3.38	0.97	0.332
Short walks around the house place [freq]:						

3 – 0	-3.82	2.51	-8.78	1.14	-1.52	0.130
4 – 0	-1.72	1.96	-5.60	2.15	-0.88	0.380
5 – 0	-0.97	1.67	-4.28	2.35	-0.58	0.565
6 – 0	-1.66	1.50	-4.62	1.30	-1.11	0.270
Long walks [freq]:						
3 – 0	1.65	0.95	-0.23	3.52	1.74	0.084
4 – 0	-0.40	1.18	-2.73	1.93	-0.34	0.737
5 – 0	0.20	1.09	-1.95	2.35	0.18	0.855
6 – 0	0.21	1.21	-2.19	2.62	0.18	0.860
Gymnastics [freq]:						
3 – 0	-0.40	1.79	-3.94	3.14	-0.23	0.822
4 – 0	0.05	1.39	-2.70	2.80	0.04	0.971
5 – 0	-0.08	0.97	-2.00	1.83	-0.09	0.930
6 – 0	0.23	0.90	-1.55	2.01	0.26	0.796
Cycling [freq]:						
3 – 0	-1.05	1.63	-4.26	2.17	-0.64	0.522
4 – 0	-0.97	1.55	-4.05	2.10	-0.63	0.532
5 – 0	1.01	1.21	-1.40	3.41	0.83	0.409
6 – 0	0.63	1.26	-1.87	3.13	0.50	0.617
Running, jogging [freq]:						
3 – 0	1.53	4.91	-8.18	11.25	0.31	0.755
4 – 0	2.09	2.08	-2.03	6.20	1.00	0.317
5 – 0	-2.73	2.99	-8.64	3.19	-0.91	0.363
6 – 0	4.14	4.11	-4.00	12.28	1.01	0.316
Swimming [freq]:						
3 – 0	-1.51	1.41	-4.30	1.27	-1.07	0.285
4 – 0	-0.40	1.39	-3.15	2.35	-0.29	0.776
5 – 0	0.78	2.00	-3.18	4.74	0.39	0.697

Nordic walking minus walking [freq]:						
3 – 0	-0.91	1.74	-4.35	2.53	-0.52	0.601
4 – 0	0.84	1.79	-2.70	4.39	0.47	0.639
5 – 0	0.98	1.42	-1.83	3.79	0.69	0.491
6 – 0	0.35	1.75	-3.12	3.82	0.20	0.843
Dancing [freq]:						
3 – 0	-0.17	1.48	-3.11	2.77	-0.11	0.910
4 – 0	0.79	1.88	-2.93	4.51	0.42	0.674
5 – 0	1.09	2.86	-4.57	6.75	0.38	0.704
6 – 0	0.56	3.46	-6.28	7.41	0.16	0.871
Work on the plot or in the garden mushroom collection [freq]:						
3 – 0	-1.72	1.47	-4.64	1.19	-1.17	0.244
4 – 0	-0.11	1.15	-2.38	2.17	-0.09	0.927
5 – 0	0.86	1.10	-1.33	3.04	0.77	0.440
6 – 0	0.14	1.21	-2.24	2.53	0.12	0.906

[a]: *reference level, 0: never-once a year-several times per year, 3: 1-2 times per month, 4: once a week, 5: several times per week, 6: daily*

Table S10. Relationship between frequency of drinking beer to the increased vs decreased body fat

Drinking beer [freq]	Change in BF	Counts	% of Total	Cumulative %
never-once a year-several times per year	decreased	70	39 %	39 %
	increased	60	33 %	72 %
1-2 times per month	decreased	17	9 %	82 %
	increased	18	10 %	92 %
once a week	decreased	5	3 %	94 %
	increased	4	2 %	97 %
several times per week	decreased	1	1 %	97 %
	increased	5	3 %	100 %

Table S11. Relationship between frequency of drinking green or white tea to the increased vs decreased body fat

Green and/or white tea [freq]	Change in BF	Counts	% of Total	Cumulative %
never-once a year-several times per year	decreased	33	18 %	18 %
	increased	21	12 %	30 %
1-2 times per month	decreased	11	6 %	36 %
	increased	11	6 %	42 %
once a week	decreased	8	4 %	47 %
	increased	11	6 %	53 %
several times per week	decreased	17	9 %	63 %
	increased	19	11 %	73 %
daily	decreased	23	13 %	86 %
	increased	25	14 %	100 %

Table S12. Relationship between frequency of drinking coffee to the increased vs decreased body fat

Coffee [freq]	Change in BF	Counts	% of Total	Cumulative %
never-once a year-several times per year	decreased	3	2 %	2 %
	increased	8	4 %	6 %
1-2 times per month	decreased	1	1 %	7 %
	increased	5	3 %	9 %
once a week	decreased	3	2 %	11 %
	increased	5	3 %	14 %
several times per week	decreased	7	4 %	18 %
	increased	10	6 %	23 %
daily	decreased	79	44 %	67 %
	increased	59	33 %	100 %

Table S13. Relationship between frequency of sweets consumption to the increased vs decreased body fat

Sweets [freq]	Change in BF	Counts	% of Total	Cumulative %
never-once a year-several times per year	decreased	11	6 %	6 %
	increased	4	2 %	8 %
1-2 times per month	decreased	10	6 %	14 %
	increased	13	7 %	21 %
once a week	decreased	24	13 %	35 %
	increased	28	16 %	50 %
several times per week	decreased	24	13 %	64 %
	increased	24	13 %	77 %
daily	decreased	23	13 %	90 %
	increased	18	10 %	100 %

Table S14. Relationship between frequency of green/white tea and coffee consumption

Green_and/or_white_tea [freq]	Coffee [freq]	Counts	% of Total	Cumulative %
0	0	1	0 %	0 %
	3	0	0 %	0 %
	4	0	0 %	0 %
	5	6	3 %	3 %
	6	57	28 %	32 %
3	0	3	1 %	33 %
	3	3	1 %	34 %
	4	2	1 %	35 %
	5	2	1 %	36 %
	6	14	7 %	43 %
4	0	3	1 %	45 %
	3	1	0 %	45 %
	4	5	2 %	48 %
	5	0	0 %	48 %
	6	12	6 %	54 %
5	0	3	1 %	55 %
	3	2	1 %	56 %
	4	0	0 %	56 %
	5	4	2 %	58 %
	6	32	16 %	74 %
6	0	2	1 %	75 %
	3	1	0 %	75 %
	4	1	0 %	76 %
	5	5	2 %	78 %
	6	44	22 %	100 %

0: never-once a year-several times per year, 3: 1-2 times per month, 4: once a week, 5: several times per week, 6: daily

Table S15. Relationship between frequency coffee and sweets consumption frequency

Sweets [freq]	Coffee [freq]	Counts	% of Total	Cumulative %
0	0	1	0 %	0 %
	3	0	0 %	0 %
	4	1	0 %	1 %
	5	2	1 %	2 %
	6	13	6 %	8 %
3	0	4	2 %	10 %
	3	2	1 %	11 %
	4	2	1 %	12 %
	5	1	0 %	13 %
	6	17	8 %	21 %
4	0	4	2 %	23 %
	3	2	1 %	24 %
	4	3	1 %	26 %
	5	5	2 %	28 %
	6	42	21 %	49 %
5	0	1	0 %	49 %
	3	2	1 %	50 %
	4	1	0 %	51 %
	5	5	2 %	53 %
	6	45	22 %	75 %
6	0	2	1 %	76 %
	3	1	0 %	77 %
	4	1	0 %	77 %
	5	4	2 %	79 %
	6	42	21 %	100 %

0: never-once a year-several times per year, 3: 1-2 times per month, 4: once a week, 5: several times per week, 6: daily
